# Early calcitonin levels in medullary thyroid carcinoma: Prognostic role in patients without distant metastases at diagnosis

**DOI:** 10.3389/fonc.2023.1120799

**Published:** 2023-02-24

**Authors:** Clotilde Sparano, Virginia Adornato, Matteo Puccioni, Elena Zago, Giuliano Perigli, Benedetta Badii, Roberto Santoro, Mario Maggi, Luisa Petrone

**Affiliations:** ^1^ Endocrinology Unit, Department of Experimental and Clinical Biomedical Sciences ‘Mario Serio’, University of Florence, Florence, Italy; ^2^ Unit of General and Endocrine Surgery, Centre of Oncological and Minimally Invasive Surgery, Department of Surgery and Translational Medicine, University of Florence, Florence, Italy; ^3^ Head and Neck Oncology and Robotic Surgery, Department of Experimental and Clinical Medicine, University of Florence, Firenze, Italy; ^4^ Consorzio Istituto Nazionale Biostrutture e Biosistemi (I.N.B.B), Rome, Italy; ^5^ Endocrinology Unit, Medical-Geriatric Department, Careggi Hospital, Florence, Italy

**Keywords:** surgery, persistent disease, calcitonin, prognosis, medullary thyroid cancer

## Abstract

**Introduction:**

Calcitonin is the most specific marker for medullary thyroid carcinoma, thus, low detectable calcitonin values after surgery can conceal persistent disease. The present study aimed to explore the prognostic role of pre-operative and *early* calcitonin levels in patients without distant metastases at diagnosis.

**Methods:**

A retrospective cohort of patients suffering from medullary thyroid carcinoma was considered (N=55). The final disease status, i.e. *complete response* (undetectable calcitonin levels and negative radiological assessments) or *persistent disease* (detectable calcitonin levels and/or positive radiological assessments), was deduced from the last available follow-up. Pre-operative and *early* calcitonin levels (i.e. six months after surgery) have been correlated to several clinical and histological features, according to the final disease status.

**Results:**

*Persistent disease* patients showed higher pre-operative and *early* calcitonin values (p=0.028 and p<0.001, respectively), compared to *complete response* sub-cohort. Cox-regression models show that early detectable calcitonin increases up to 18-fold the risk of persistent disease, independently from tumour size and pre-operative calcitonin levels (p=0.006). Of note, when considering only patients who finally developed distant metastasis, ROC curve analysis shows that an *early* calcitonin level ≥16 pg/ml predicts the final disease status with a sensitivity of 89% and a specificity of 82% (AUC=0.911, CI95%: 0.819-1000, p<0.001).

**Conclusion:**

Calcitonin levels six months after surgery represents an easy and effective predictor of persistent disease for medullary thyroid carcinoma without distant metastasis at diagnosis.

## Introduction

Medullary thyroid carcinoma (MTC) represents a rare and potentially aggressive thyroid cancer histotype, arising from the neuroendocrine parafollicular C-cells. Compared to follicular thyroid neoplasms, MTC accounts for only 5% of all thyroid cancers, and, despite the usually long survival (1), it is characterized by a high rate of metastasis to lymph nodes (from 50% to 90% of cases) (2–4) and by a prevalence of 10% of distant metastases, at diagnosis (1).

Although the large majority of cases are sporadic, up to 25% of MTC disclose a genetic predisposition, due to a germline *RET* gene mutation, which also drives other endocrinological tumours in the framework of the syndrome Multiple Endocrine Neoplasia type 2 (MEN2) (1). Out of syndromic MTC, molecular studies showed somatic *RET* mutations in almost 80% of cancer samples (5), resulting in more aggressive clinical behaviour. Radioiodine therapy is ineffective in MTC, while surgical radicalization, coupled with an extended lymph node resection, is considered the primary therapeutic option for this cancer (1, 2).

In the last years, several advancements have been made in terms of systemic therapies, thanks to the approval of large spectrum tyrosine-kinase inhibitors at first (vandetanib and cabozantinib) (6, 7), followed by direct anti-RET drugs, which recently entered within the therapeutic panorama (8). However, targeted therapies are intended for patients with progressive disease and higher metastatic load, to prolong survival and to reduce tumour-related adverse events. In contrast, the large majority of MTC patients undergo a long biochemical and instrumental follow-up, without active therapy. On the other hand, fewer advancements have been made in monitoring tools and in detecting new prognostic factors. The traditional management of MTC still relies on the serial measurement of calcitonin (CT) and carcinoembryonic antigen (CEA) and their doubling times, whose increase suggests disease progression and/or metastatic spread (1). In particular, CT determinations represent the most specific tumour marker for MTC, but their measurement is still influenced by differences in evaluation timing and by predictive cut-offs. Modern immunochemiluminometric assays have narrowed some analytical issues, making CT measurement more reliable and accurate (1). Metastatic disease is associated with higher CT levels and unfavourable outcome. In fact, according to current guidelines (1), there is a consensus about a negative prognostic value of high post-operative CT levels - i.e. more than 150 pg/ml - while no evidence is available about early detected lower levels.

Post-operative CT requires from a few days to several months to reach its nadir (1, 9). For this reason, several CT evaluations are necessary to catch the lowest value, which will be the reference during the follow-up. In the absence of clinically evident metastatic lesions, low CT levels are not necessarily related to MTC, but they can conceal a persistent disease, irrespective of negative radiological assessments.

The present study aims to evaluate potential prognostic factors in a retrospective monocentric cohort of sporadic and syndromic MTC with variable follow-up lengths. To identify early and easier predictors of persistent disease, we specifically focused on the predictive role of pre-operative and early post-operative CT levels (i.e. at six months follow-up), exploring their relationship to the outcome and their ability to individuate cases requiring additional monitoring. Finally, to better evaluate the role of CT levels without known clinical interferences, we excluded patients with distant metastases at diagnosis.

## Materials and methods

The present retrospective study considered a consecutive series of adult patients referred to the Endocrine Unit of Careggi Hospital from February 2003 to February 2022, and who provided an informed consent. Inclusion criteria are: i) total thyroidectomy with a diagnosis of MTC on histology; ii) availability of histological, clinical and biochemical data; iii) absence of distant metastasis (M0) at diagnosis (by negative preoperative thoracic and abdomen computer tomography evaluation). Exclusion criteria are: i) absence of histological or biochemical information; ii) follow-up performed outside from the Endocrine Unit of Careggi Hospital; iii) presence of significant comorbidities (i.e. chronic renal failure) and/or ongoing medications interfering with CT assessments (i.e. pump proton inhibitors).

For each patient, we collected all clinical (gender, age at diagnosis, follow-up length), histological (including tumour size, multifocality, vascularization, number of metastatic lymph nodes at diagnosis), biochemical (CT and CEA measurements at diagnosis and during follow-up) and radiological information. In particular, neck ultrasound (US) results were available for 79% and 77% of cases at six months and one-year follow-up, respectively.

Biochemical tests have been performed in Careggi Hospital and CT measurement has been performed by chemiluminescence immunoassay LIAISON^®^ XL (DiaSorin).

According to the results of the last available follow-up, each patient has been classified as *complete response* (CR) if: undetectable CT values (CT values below the lower reference limit of 0.1 pg/ml), normal CEA values and negative radiological assessment; *persistent disease* (PD) if: detectable serum CT and/or radiological evidence of diseases.

All histology has been classified according to the AJCC VIII edition (10). Germline and/or somatic assessment of RET mutations have been collected, when available (91% and 51%, respectively).

The present study was approved by the Local Ethics Committee (Comitato Etico Area Vasta Centro-CEAVC, Florence, Tuscany, Italy) and conducted in compliance with the Declaration of Helsinki principles.

### Statistical analysis

Continuous variables have been expressed as mean ± standard deviation (SD) if normally distributed or as median [quartile] when non-normally distributed. Categoric variables have been expressed as numbers and percentages. A *complete response* (CR) and *persistent disease* (PD) dummy variable have been built from patients’ outcomes at the last available follow-up.

According to the sample size, differences between the two subgroups were analysed using the T-test or Mann-Whitney test for continuous variables and the Chi-square test or Fisher-Yates test for dummy variables. The Receiver Operating Characteristic (ROC) curve analysis was applied to calculate threshold values, sensitivity and specificity. For each threshold, positive (PPV) and negative predictive values (NPV) have been calculated. According to the available sample size, a Cox proportional hazard regression model with stepwise selection based on the Akaike Information Criterion (AIC) has been used to select the best-fitting model, considering the most significative variables, and using the patients’ final disease status as the dependent variable. All the analysis has been performed using IBM SPSS Statistics for Windows version 28.01.0 (142), R version 4.1.2 (2021–11–01), and GraphPad Prism version 9.0.0 for Windows, GraphPad Software, San Diego, California USA, www.graphpad.com.

## Results

### Population sample

From a total number of 60 available patients, 55 met the inclusion criteria and were considered in the final analysis. Thereafter five patients were excluded, because of the absence of required information. Considering the final outcome, patients with either biochemical (N=13) or metastatic disease [locoregional lymph nodes (LN) and/or distant metastases] (N=14) at the last available follow-up, have been considered as *persistent disease* (PD) patients (N=27). **Table 1** shows an overview of the main population features, according to their final disease status (i.e. CR or PD). Briefly, 45% of the patients (25) were male with a mean age of 59 years, both without differences between CR and PD patients. Initial surgery included total thyroidectomy and 38 (67.9%) central compartment dissections, three (5.4%) central and right lateral-cervical dissections (levels II-V) and seven (12.5%) central and bilateral cervical dissections (levels II-V). No lymph node dissection was performed for eight cases (14.3%) due to negative preoperative investigations and sub-centimetric thyroid nodules. Among them, six patients disclosed incidental MTC and all belonged to the CR cohort (p=0.023). In ten cases (18%), concurrent differentiated thyroid cancer was present, without significant differences between groups CR and PD (p=0.446).

**Table 1:** Patients’ overview according to disease status.

Factor	Group	Final disease status*		p-value
		Complete response	Persistent disease	
		Total=28	Total=27	
**Gender (%)**	Female	19 (67.9)	11 (40.7)	0.060
	Male	9 (32.1)	16 (59.3)	
**Age (years)**	Mean (SD)	58.63 (16.18)	60.15 (14.54)	0.720
**Germinal RET mutation (%)**	Absent	21 (75.0)	21 (77.8)	0.150
	Present	6 (21.4)	2 (7.4)	
	Unknown	1 (3.6)	4 (14.3)	
**Somatic RET mutation (%)**	Absent	9 (32.1)	9 (33.3)	0.291
	Present	2 (7.1)	8 (29.6)	
**Tumour size (mm)**	Median (IQR)	10.00 [6.00, 19.50]	22.00 [13.00, 31.00]	**0.002**
**Vascular invasion (%)**	Absent	17 (60.7)	11 (40.7)	**0.018**
	<4 foci	0 (0.0)	3 (11.1)	
	>4 foci	0 (0.0)	2 (7.5)	
	Unknown	11 (39.3)	11 (40.7)	
**Multifocality (%)**	Absent	21 (75.0)	8 (29.6)	**0.005**
	Present	7 (25.0)	15 (55.6)	
	Unknown	0	4 (14.8)	
**Incidental (%)**		6 (20.7)	0 (0.0)	**0.023**
**T (%)**	T1a	15 (51.7)	4 (16.0)	**0.023**
	T1b	6 (20.7)	5 (20.0)	
	T2	7 (24.1)	10 (40.0)	
	T3	1 (3.4)	5 (20.0)	
	T4	0 (0.0)	1 (4.0)	
**N (%)**	N0	18 (64.3)	7 (25.9)	**0.001**
	N1a	3 (10.7)	8 (29.6)	
	N1b	1 (3.6)	10 (37.0)	
	Nx	6 (21.4)	2 (7.4)	
**STAGE**	I	18 (64.4)	4 (14.8)	**<0.001**
** **	II	6 (21.4)	5 (18.5)	
** **	III	2 (7.1)	7 (25.9)	
** **	IVA	2 (7.1)	11 (40.7)	
**Preoperative CT**	Median (IQR)	122.00 [72.70, 460.00]	1016.50 [213.10, 2000.0]	**0.028**
**Preoperative CEA**	Median (IQR)	7.95 [4.05, 13.82]	34.00 [15.00, 67.50]	**0.011**
**CT at 6 months FU**	Median (IQR)	0.00 [0.00, 0.00]	25.40 [4.88, 346.25]	**<0.001**
**CEA at 6 months FU**	Median (IQR)	2.20 [1.17, 2.45]	4.95 [2.25, 6.65]	**0.01**
**CT at one-year FU**	Median (IQR)	0.00 [0.00, 0.00]	31.75 [5.50, 431.25]	**<0.001**
**CEA at one-year FU**	Median (IQR)	1.45 [1.00, 3.25]	4.50 [1.38, 7.35]	0.093
**Dead (%)**		1 (3.4)	5 (19.2)	0.101

Bold numbers highlight significant p-values.

*Final disease status has been determined according to the last available follow-up.

CT, calcitonin; CEA, carcinoembryonic antigen; FU, follow-up; SD, standard deviation; IQR, interquartile range.

No significant differences were also observed in germinal or somatic molecular status. In particular, six CR and two PD patients disclosed germinal RET mutations (p=0.150), while two CR and eight PD disclosed somatic RET mutations (p=0.291) (**Table 1**). Considering histological features, differences in tumour size (p=0.002), vascular invasion (p=0.018) and multifocality (p=0.005) were observed between CR and PD.

At diagnosis, PD patients were characterized by a wider LN involvement (p=0.001) and by a more advanced stage (p=0.003), with 41% of cases classed as IVA stage disease (**Table 1**). In particular, 64% of CR patients were classed as N0 and the number of metastatic LN ranged from zero to two, at odds with PD patients, who were classed as N0 in the 26% of cases and showed a median number of four metastatic LN [0-41] (p<0.001) (**Table 1**).

After a median follow-up of 39.2 months [IQR: 16.3-83.7], 12 patients (21%) developed distant metastases, while six patients died, with non-significant differences between groups, i.e. one and five cases for CR and PD patients, respectively. Of note, the CR patient’s death was allocated to cardiovascular disease and, therefore, his death was not apparently related to MTC. In fact, at the last available follow-up, he was both radiologically and biochemically negative. Considering the metastatic patients, in half of the cases (50%) the initial recurrence was entirely out of the neck, i.e. sited in the liver, lungs and/or bone.

### Biochemical and ultrasound features

**Figure 1:**
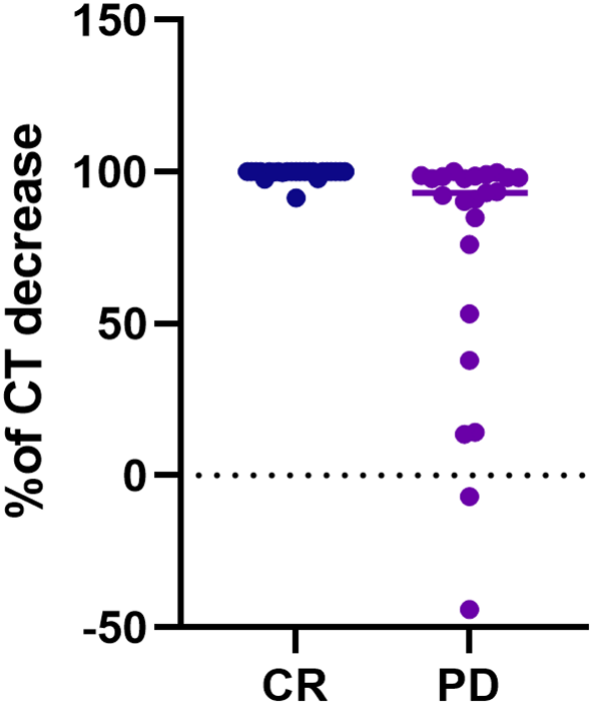
Scatter plot of the CT decreasing rate (%) from pre-operative values to the six months levels, according to the final disease status, i.e. complete response vs persistent disease. The final disease status was calculated considering the disease status at the last available follow-up. CT, calcitonin; CR, complete response; PD, persistent disease.

**Figure 2:**
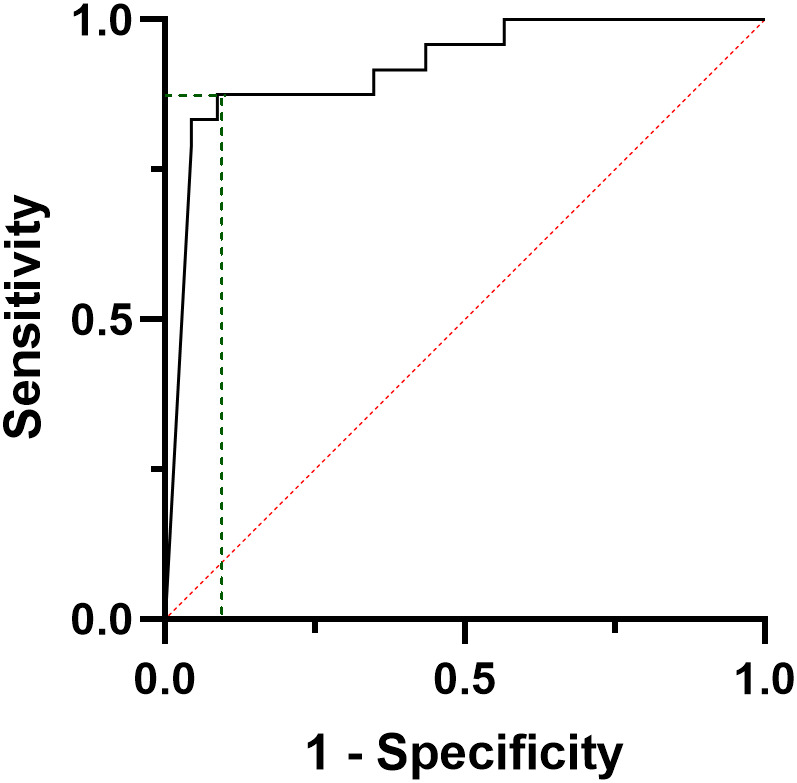
ROC curve analysis considering the decreasing rate of calcitonin values from the baseline to the six months after surgery, according to the final disease status, i.e. complete response vs persistent disease.

Considering biochemical features, several differences were observed. PD patients showed higher CT and CEA values at pre-operative assessments (p=0.028 and p=0.011, respectively), and at post-surgical follow-up, i.e. six months (p<0.001 and p=0.010, respectively), while at one-year follow-up only CT levels resulted significantly different between groups (p<0.001) (**Table 1**). Considering the decreasing rate of CT levels from baseline (**Figure 1**), the accuracy of the relationship was 0.921 (CI95%:0.84-1.00, p<0.0001) (**Figure 2**). When CT level was reduced up to 99.4% the sensitivity and specificity for the final disease status were 87.5% and 91.3%, respectively. When CT level was reduced to 95% the sensitivity was very high (95.8%), but the specificity decreased to 56.5%. For the 95% cut-off, the PPV and NPV were 92.9% and 69.7%. For the 99.4% cut-off, the PPV and NPV were 91.3% and 87.5%, respectively. When both cut-offs of CT reduction (i.e. 99.4% and 95%) were iteratively introduced in a Cox regression analysis along with tumour size, multifocality (yes/no) and presence of positive lymph nodes (yes/no), the two cut-offs retained significance in predicting the final disease status [Exp(B)=0.108 (CI95%:0.022-0.525), p=0.006 and Exp(B)=0.241 (CI95%:0.086-0.678), p=0.007, respectively]. Interestingly, the other putative predictive factors lost significance when CT was reduced by 99.4%, while only tumour size remain significant (p=0.033) when CT was reduced by 95%.

Regarding US results, at six months only one CR and three PD patients showed doubtful findings, while none of the CR and five PD patients showed persistent disease (i.e. positive LN). At the one-year evaluation, 11 CR patients showed negative US imaging.

### Prognostic analysis

**Figure 3:**
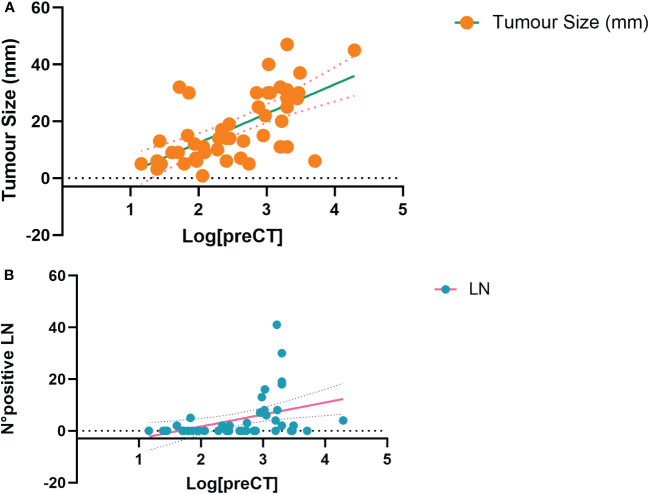
Curve estimation plots show the linear relationship, between Log preoperative CT levels and both tumour size (F=31.22, p<0.001, **A**) and the number of positive lymph nodes (F=8,14, p=0.007, **B**). Log, logarithmic; CT, calcitonin; N°, number; LN, lymph nodes.

Considering pre-operative CT, a series of curve regression modelling was performed to explore the potential correlation between tumour size and the number of pathologic LN. **Figure 3** shows, as bubble plots, the linear correlation between preoperative Log[CT] and both tumour size (F=31.22, p<0.001) (**Figure 3A**) and the number of metastatic LN (F=8,14, p=0.007) (**Figure 3B**).

**Figure 4:**
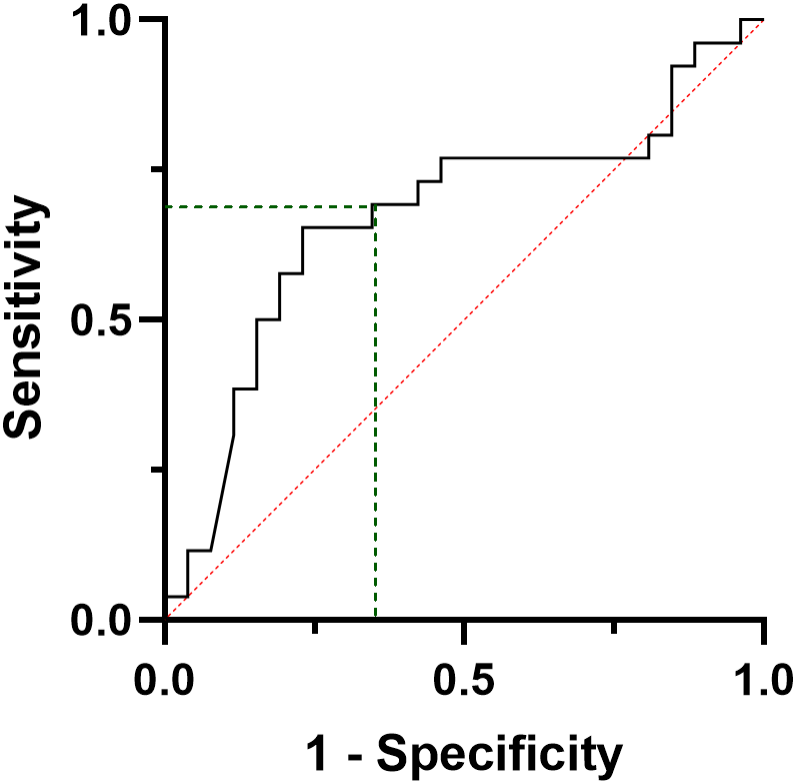
ROC curve analysis considering the preoperative calcitonin values, according to the final disease status, i.e. complete response vs persistent disease.

ROC curve analysis was performed to find the best CT threshold able to predict the final outcome at pre-operative evaluation (**Figure 4**). A pre-surgical CT value of 284 pg/ml showed a sensitivity and a PPV of 69.2%, a specificity and NPV of 68.0% in predicting the final outcome (AUC= 0.679, CI95:0.525-0.833, p=0.028) (**Figure 4**). In a similar ROC model, the lowest detectable CT value at six months follow-up (i.e. 0.7 pg/ml) of the present cohort was able to predict the final outcome with a sensitivity and a specificity of 95.8% and 80.0%, respectively (AUC= 0.958, CI95: 0.900-1.0, p<0.001) and a PPV and NPV of 82.1% and 95.2%, respectively.

In particular, only five patients (9.1% of the population) with a final CR status showed a detectable CT value at the six-month follow-up (from 0.7 to 5.69 pg/ml), which gradually become undetectable.

Considering these results, a dummy variable of *early detectable* CT (yes/no) was built, according to the detectable or undetectable status of the six-month CT levels. Thereafter, a Cox proportional hazard regression analysis has been performed, using a stepwise selection based on AIC and the final disease status as readout. Due to the present population size (N=55), five simultaneous putative factors have been introduced, i.e. the two CT thresholds, the tumour size, the presence of lymph node metastases (yes/not), and the multifocality (yes/not). **Table 2** shows that when including all these predictors, the best-fitting model finally retains only three prognostic factors, i.e. the tumour size, the *early detectable* CT, and the preoperative CT threshold ≥284 pg/mL. However, only the *early detectable* CT resulted significantly and independently associated with the final disease status, with an OR=18.53 (CI95%: 2.25-152.80, p=0.00669).

**Table 2:** Cox proportional hazard regression model by stepwise selection based on AIC, considering the most performing prognostic factors and using the final disease outcome as readout.

	OR	Confidence interval 95%		p-value
		Lower	Upper	
**Early detectable CT**	18.53	2.25	152.8	**0.00669**
**Preoperative CT ≥284 pg/ml**	0.36	0.1	1.29	0.1164
**Tumour size (mm)**	1.03	0.99	1.07	0.06956

Bold numbers highlight significant values.

OR, odds ratio; CT, calcitonin.

**Figure 5:**
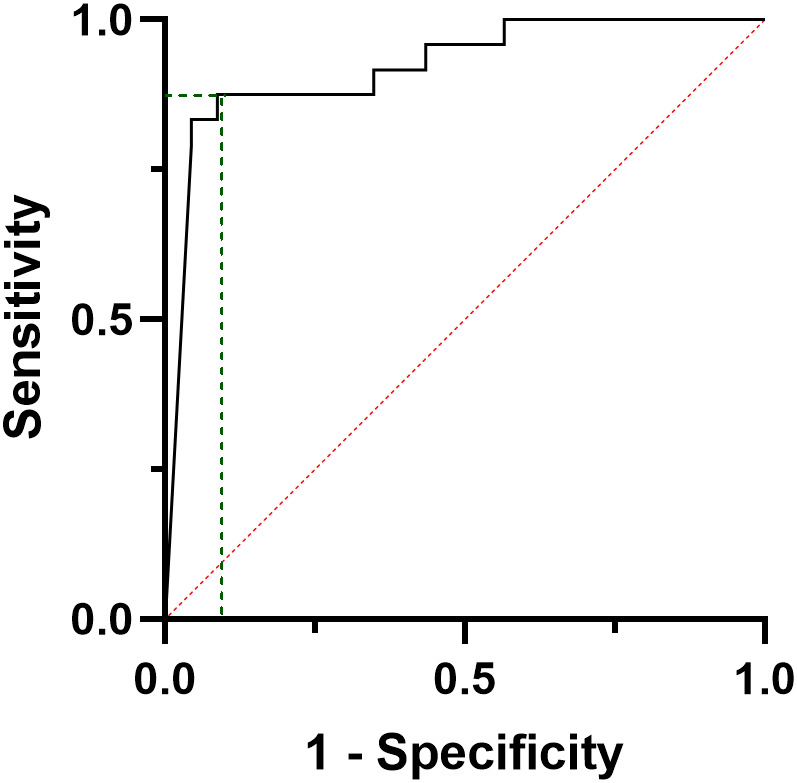
ROC curve analysis considering the calcitonin values at six months post-surgical evaluation of patients developing distant metastases during follow-up.

In a sub-analysis, when only metastatic (M1) patients at the final follow-up were considered (N=12), a ROC curve analysis shows that a 16 pg/ml six-month CT was able to predict the development of distant metastasis with a sensitivity of 89% and a specificity of 82% (AUC=0.911, CI95%: 0.819-1000, p<0.001) and a PPV and a NPV of 100% and 73.5%, respectively (**Figure 5**).

## Discussion

The present study shows that an early CT evaluation at six months of follow-up is yet informative and cost-effective to predict the final disease status in a cohort of MTC patients without distant metastases at diagnosis. A timely decrease of CT levels of 99.4% from the baseline, reduces by nearly 90% the risk of persistent MTC, regardless of other known prognostic predictors. Furthermore, an *early detectable* CT value, i.e. any detectable CT value after six months from surgery, appears as a reliable alert of persistent disease, increasing by 18-fold the risk of recurrence, independently from the preoperative CT levels and the tumour size. Conversely, pre-operative CT levels were confirmed as associated with the tumour size and with the number of metastatic lymph nodes, but were less suited to estimate the medium-to-long-term disease status.

Despite the advantages in terms of systemic treatments, the traditional follow-up for MTC has not yet moved forward. It is recognized that undetectable postoperative CT values represent a favourable predictor of disease recovery (1, 9, 11–13). Conversely, the presence of distant metastases, even if infrequent at diagnosis, has a known negative prognostic value and it is associated with higher postoperative levels of CT (1). Between these extremes, a significant proportion of patients disclose a variable range of detectable low CT levels, falling within a wider field of prognostic uncertainty (14). CT is still the most specific marker of MTC, but the efforts in optimizing its sensitivity didn’t produce significant results. CT doubling time is advocated as an alert for progressive disease (1) when the marker swiftly increases in less than six months, but it requires subsequent CT evaluations over time and, accordingly, it doesn’t represent a systematic early predictor.

To date, several studies explored potential prognostic factors for MTC. According to those studies, the presence of lymph node involvement, distant metastasis and the disease extension at diagnosis were unanimously recognized as unfavourable prognostic factors (11, 15–20). On the other hand, the significance of clinical features, such as age and gender, are more controversial, at odds with differentiated thyroid cancer. Only one study on a small cohort of 31 patients showed a greater incidence of MTC in female patients (17). The role of age is even more controversial. Older patients appear to suffer from a worse prognosis, but with discordant results and without an accepted cut-off (17, 21, 22).

From a biochemical perspective, the role of early post-operative CT patients has not been fully explored. The available data are from relatively old studies, often biased by several methodological issues. For instance, Modigliani et al. (11) analysed a wide sample of 889 MTC from a French database of the French Calcitonin Tumors Study Group (GETC). The Authors found that only age and stage resulted as significant prognostic factors, while biochemical cures (i.e. normalization of CT and CEA values) predict about 98% of 10-year survival (11). In addition, different methods for CT evaluation were used, according to the centres and the period of diagnosis. Finally, in Modigliani et al. study no specific data about six months postoperative CT have been provided. A 2020 study by Kotwal et al. (16) on 163 MTC patients observed that, when the post-operative CT is detectable, the overall survival decreases with an HR of 1.00 for every 100 pg/ml of CT. Once again, no specific information on 6 months’ CT levels, nor cut-offs were provided; furthermore, this population included a significant proportion of M1 patients, as opposed to the present study (16). The importance of early disease evaluation has been observed in a multicentre study on 193 MTC (18), where the disease status (recovered/persistence) at one-year was associated with the one at the last available follow-up. The Authors found that the M1 status and the extra-thyroid extension at diagnosis were predictors of recurrence, but no biochemical data were analysed (18).

Current guidelines suggest performing an early CT evaluation three months after surgery (1). However, this timing may be too premature to evaluate the CT nadir, since different CT isoforms or aggregates from tumour deposits may persist in the bloodstream and require additional time to be cleared (9). The present study is the first to explore the clinical value of an *early* six months CT evaluation in patients with only locoregional disease at diagnosis, thus virtually eligible to surgical radicalization and biochemical cure. In line with the former evidence (11, 15–20), patients’ long-term prognosis is favourable when a timely biochemical cure is reached. On the other side, in non-M1 patients, the persistence of low detectable CT values, irrespective of their raw levels, may hide persistent disease and identify a potential intermediate-risk subgroup, requiring additional monitoring. In fact, in the present study, only less than 10% of patients with low CT levels at six-month evaluation showed a final CR at the end of available follow-up. The current guidelines suggest performing further radiological investigations when CT levels are >150 pg/ml (1). The present results are substantially in line with this indication since lower levels of CT often do not correspond to an evident morphologic disease. However, the early evaluation of CT values seems to be yet reliable of higher risk of recurrence and might select a specific subset of patients to be more strictly observed, for a concrete risk of metastatic disease.

We recognize that the sample size, the retrospective design and the monocentric population represent the main weaknesses of the present analysis. However, the present results are interesting because: they investigated a selected and rare population, with uniform clinical, biochemical and histological examinations and a valid medium- to long-term follow-up. In fact, the medium follow-up was about three years and 29% and 16% of the population was followed up for five and ten years, respectively.

## Conclusion

MTC is a rare and insidious tumour, where prognostic and effective predictive tools are still tough to be found. An early six months CT evaluation may represent a useful and economic screening test for sporadic and familiar MTC populations, without distant metastases at diagnosis, but at risk of persistent/recurrent disease. Whether confirmed in a wider and multicentre prospective population, an early CT *alert* threshold could identify a subset of the population, who need awareness during follow-up.

## Data availability statement

The raw data supporting the conclusions of this article will be made available by the authors, without undue reservation. The access is available upon specific request to the corresponding author due to privacy restrictions.

## Ethics statement

The studies involving human participants were reviewed and approved by Comitato Etico Area Vasta Centro-CEAVC, Florence, Tuscany, Italy. The patients/participants provided their written informed consent to participate in this study.

## Author contributions

Conceptualization, CS. Data collection, EZ, VA and MP. Writing original manuscript and performed statistical analysis CS, LP and MM. Review and editing, CS, LP, MM, EZ, VA, MP, GP, BB and RS. All authors contributed to the article and approved the submitted version.
